# Polycystic ovary syndrome phenotype does not have impact on oocyte morphology

**DOI:** 10.1186/s12958-021-00874-2

**Published:** 2022-01-05

**Authors:** Audrey Uk, Christine Decanter, Camille Grysole, Laura Keller, Hélène Béhal, Mauro Silva, Didier Dewailly, Geoffroy Robin, Anne-Laure Barbotin

**Affiliations:** 1 CHU Lille, Institut de Biologie de la Reproduction-Spermiologie-CECOS, Jeanne de Flandre Hospital, Lille, France; 2 Inserm UMR-S 1172, Laboratory of Development and Plasticity of the Neuroendocrine Brain, Lille, France; 3 Department of Medicine, University of Lille, Lille, France; 4 CHU Lille, Department of Endocrine Gynecology and Reproductive Medicine, Jeanne de Flandre Hospital, Lille, France; 5 Inserm EA 4308 Gametogenèse et Qualité du Gamète, Institut de Biologie de la Reproduction-Spermiologie-CECOS, Lille, France; 6 Univ. Lille, CHU Lille, ULR 2694 - METRICS: Évaluation des Technologies de Santé et des Pratiques Médicales, Lille, France

**Keywords:** Polycystic ovary syndrome, PCOS phenotypes, Oocyte morphology, Morphologic scores, ICSI

## Abstract

**Purpose:**

The primary objective of the present study of women participating in an ICSI program was to determine whether the morphologic quality of oocytes was related to the polycystic ovary syndrome (PCOS) phenotype.

**Methods:**

We performed a retrospective cohort study in the IVF unit at the Lille University Medical Center (Lille, France) between 2006 and 2015. Oocyte morphology (fragmented first polar body, abnormal zona pellucida, large perivitelline space, material in perivitelline space, abnormal shape of oocyte, granular cytoplasm and intracytoplasmic vacuoles) was evaluated in PCOS women and according to different subgroup (depending on the presence or absence of the cardinal features polycystic ovarian morphology (PCOM), hyperandrogenism (HA), and oligo-anovulation (OA)).

**Results:**

A total of 1496 metaphase II oocytes (*n* = 602 for phenotype A combining PCOM + HA + OA, *n* = 462 oocytes for phenotype C: PCOM + HA, and *n* = 432 for phenotype D: PCOM + OA) were assessed. The phenotypes A, C and D did not differ significantly with regard to the proportion of normal oocytes (adjusted percentages (95%CI): 35.2% (31.5 to 39.1%), 25.8% (21.9 to 29.9%) and 34.0% (29.7 to 38.6%), respectively: adjusted *p* = 0.13). Likewise, there were no significant intergroup differences in oocyte morphology. The ICSI outcome was not significantly associated with the PCOS phenotype.

**Conclusion:**

The present study is the first to show that the PCOS phenotype (notably the presence vs. absence of OA and/or HA) is not significantly associated with the morphological quality of oocytes.

**Supplementary Information:**

The online version contains supplementary material available at 10.1186/s12958-021-00874-2.

## Introduction

Polycystic ovary syndrome (PCOS) is the most common endocrine reproductive disorder; worldwide, it affects 5-20% of women of reproductive age [1]. According to the Rotterdam consensus criteria and after the exclusion of related disorders, the diagnosis of PCOS is based on the presence of at least two the following cardinal features: oligo-ovulation/anovulation (OA), clinical and/or biochemical hyperandrogenism (HA), and a polycystic ovarian morphology (PCOM) on ultrasound with > 20 follicles per ovary [2–4]. Depending on the presence or absence of these cardinal features, a case of PCOS is then classified as phenotype A, B, C or D [2, 5]. PCOS phenotype A is characterized by the presence of all three features (i.e. OA + HA + PCOM), whereas the other phenotypes are characterized by the presence of only two of the features.

In vitro fertilization (IVF) may be required for women with anovulatory PCOS and who have failed to become pregnant with ovulation induction or when there are additional infertility factors, such as tubal stenosis or male subfertility [4, 6, 7]. It has been postulated that PCOS women produce more oocytes than healthy women in response to controlled ovarian hyperstimulation (COH) [8–11]. However, it is also suspected that these PCOS oocytes may be of poor quality as a result of intra- and extra-ovarian factors [12]; this might lead to a lower fertilization rate, poor embryo quality, a lower implantation rate and a higher miscarriage rate [8–10, 13, 14]. Indeed, it is well known that oocyte competence influences embryonic development [15]. To date, only a few studies have assessed the potential impact of different PCOS phenotypes on the outcomes of assisted reproductive technologies [16–19]. In a study of IVF procedures, Ramezanali and colleagues (2016) reported lower clinical pregnancy rates in PCOS phenotypes A and B than in controls - suggesting that the combination of HA and OA may affect embryonic development [18]. Furthermore, the risk of miscarriage was greater for phenotypes A, B and D than for phenotype C (ovulatory PCOS) [16]. The heterogeneity of the PCOS study populations in these reports (i.e. interstudy differences in the inclusion criteria and a mixture of IVF and ICSI procedures) makes it difficult to draw definitive conclusions. Nevertheless, it is likely that the oocytes are of poor morphologic quality in cases of full-blown PCOS. Furthermore, several studies have found that the oocyte’s developmental competence might be impaired in women with PCOS [12, 20]. In particular, it has been suggested that the oocyte’s morphologic quality is linked to its implantation potential [21, 22]. On the one hand, Alikani and colleagues reported reduced pregnancy and implantation rates in women with exclusive replacement of embryos, originating from dysmorphic oocytes regardless of the oocyte abnormality features [21]. On the other hand, Kahraman and colleagues have suggested that the oocytes with severe central granulation in their cytoplasm seem to have less implantation and on-going pregnancy potential [22]. Oocyte quality is a key limiting factor in female fertility; it reflects the oocyte’s intrinsic developmental potential and has an essential role in fertilization and subsequent development [23].

An oocyte’s quality is primarily reflected by its morphologic features. For instance, centrally located cytoplasm granulations are associated with low fertilization rates [24], poor pregnancy outcomes, and low live birth and miscarriage rates in ICSI programs [25]. However, little is known about the relationship between oocyte morphology and the PCOS phenotype. Indeed, the influence of the oocyte’s competence on the reproductive potential of women with PCOS depends on the phenotype and the related comorbidities [15]. Thus, the primary objective of the present study of women in an ICSI program was to determine whether oocyte morphologic quality varies from one PCOS phenotype to another. To this end, we used the criteria routinely applied in IVF centers to evaluate the oocytes’ morphological features. The study’s secondary objective was to determine whether the oocyte morphology differs when comparing women with PCOS, women with PCOM only, and other (control) women in an ICSI program.

## Material and methods

### Study design and population

Data on clinical, endocrine and ultrasound features were extracted from a computerized database compiled between January 2006 and December 2015 in the IVF center at Lille University Medical Center (Lille, France). Women having participated in an ICSI program were included retrospectively. The study results were reported in compliance with the STROBE statement [26]. In line with the French legislation on retrospective studies of routine clinical practice, the study protocol was approved by the Lille hospital committee.

For the PCOS group, the inclusion criteria were (i) age 18–38, (ii) a confirmed diagnosis of PCOS (see below); (iii) the presence of at least one COH procedure followed by successful oocyte retrieval; and (iv) inclusion in an ICSI program in a sole indication of male infertility. The non-inclusion criteria were (i) previous pelvic or ovarian surgery, (ii) previous or ongoing endocrine disorders (other than PCOS), and (iii) use of medications known to have an influence on the endocrine profile. The following exclusion criteria were: age < 18 or more than 38 years, a low suspected ovarian reserve [27] (FSH > 12 IU/l), pregnant or breastfeeding women, and hormonal contraceptive users. We excluded women with differential diagnosis for PCOS: hyperprolactinemia (serum prolactin > 20 ng/ml on two separate determinations), functionnal hypothalamic amenorrhea, other congenital or acquired gonadotropic deficiencies, nonclassic 21-hydroxylase deficiency (basal 17-hydroxyprogesterone (17-OHP) > 5 ng/ml and/or post-adrenocorticotrophic hormone-stimulated value > 12 ng/ml). Ovarian or adrenal tumours were excluded on the basis of serum total testosterone (TT) or dehydroepiandrosterone sulfate levels lower than 1.5 ng/ml or 15 μmol/l, respectively. Moreover, any patient with at least one follicle with a diameter > 9 mm at U/S or a serum estradiol (E2) level above 80 pg/ml was excluded from the study. Women with a history of pelvic or ovarian surgery, severe endometriosis, anovulation and/or hyperandrogenism not related to PCOS were also excluded from the study.

All patients were diagnosed with PCOS (according to the modified Rotterdam classification) and so fulfilled at least two of the following three criteria: OA, clinical and/or biochemical HA, and PCOM (defined as ≥19 follicles per ovary) and/or an excessive serum anti-Müllerian hormone (AMH) level ≥ 35 pmol/l), as previously reported [28, 29]. Clinical HA was defined as the presence of hirsutism (with a modified Ferriman–Gallwey score ≥ 6) and/or acne in more than two body areas [30]. Biochemical HA was defined as serum total testosterone (T) and/or androstenedione level above the 95th percentile value for controls with normal cycles (> 0.53 ng/mL and > 2.07 ng/mL, respectively) [29]. OA was defined as amenorrhea or a cycle length above 35 days.

We included a total of 65 women in the PCOM-only group. Indeed, some researchers consider that PCOM is part of a complex PCOS spectrum disorder, with abnormal granulosa cell activity [31, 32]. The inclusion criteria for this group were (i) age 18–38, (ii) PCOM defined as either an ovarian volume ≥ 10 ml, an ovarian surface area ≥ 5.5 cm^2^, a follicle number per ovary (FNPO) ≥19 or an AMH level ≥ 35 pmol/l or both [28], (iii) the presence of at least one COH procedure followed by successful oocyte retrieval; and (iv) inclusion in an ICSI program in a sole indication of male infertility. The following exclusion criteria were: age < 18 or more than 38 years, a low suspected ovarian reserve [27], pregnant or breastfeeding women, any additional Rotterdam criteria (i.e. HA and/or OA), hormonal contraceptive users, previous pelvic or ovarian surgery and any risk factors for infertility (pelvic surgery, endometriosis etc) and use of medications known to have an influence on the endocrine profile.

For the control group comprising 58 women, the inclusion criteria were (i) age 18–38, (ii) ovulatory cycles, no signs of hyperandrogenism, an FNPO between 8 and 18 follicles in each ovary, FSH < 10 IU/L, and AMH < 35 pmol/L, (iii) the presence of at least one COH procedure followed by successful oocyte retrieval; and (iv) inclusion in an ICSI program in a sole indication of male infertility. The following exclusion criteria were: age < 18 or more than 38 years, a low suspected ovarian reserve [27], pregnant or breastfeeding women, and hormonal contraceptive users, (previous pelvic or ovarian surgery, any Rotterdam criteria or risk factors for infertility (pelvic surgery, endometriosis etc.) and use of medications known to have an influence on the endocrine profile.

The 110 women diagnosed with PCOS were classified into four phenotype subgroups (Table 1). As there was only one woman with a PCOS B phenotype, we excluded her (for reasons of representativeness) from the study population. Hence, 109 women were included in the final analysis (PCOS A: 41 patients and 77 cycles; PCOS C: 31 patients and 57 cycles; PCOS D: 37 patients and 55 cycles). A total of 189 ICSI treatment cycles were analyzed during the study period. In all cases, male factor infertility indicated ICSI with fresh ejaculated spermatozoa only. ICSI cycles using surgically acquired sperm were excluded. Additionally, only ICSI cycles following sperm processing using density gradient for sperm selection were included in the study.

**Table 1:** Criteria defining the four PCOS phenotypes

	Polycystic ovary syndrome			
Modified Rotterdam Criteria	Phenotype A	Phenotype B	Phenotype C	Phenotype D
Oligoanovulation	Yes	Yes	No	Yes
Clinical and/or biochemical hyperandrogenism	Yes	Yes	Yes	No
PCOM, AMH ≥35 pmol/l, or both	Yes	No	Yes	Yes

### Laboratory tests and ultrasound examinations

All patients had a baseline endocrine screen and ultrasound examination on the same day during the early follicular phase (i.e. between days 2 and 5). In patients with OA, the menstrual period was either spontaneous or induced by the administration of dydrogesterone. Serum FSH and estradiol (E2) levels were measured using chemiluminescent, two-site immunoassays on a multiparameter system (Axsym®; Abbott Laboratories, Chicago, IL, USA). Serum AMH levels were measured using a second-generation enzyme immunoassay (A16507, Beckman Coulter, Immunotech, Villepinte, France). Delta-4-androstenedione and total testosterone levels were measured in duplicate using a radioimmunoassay, as described previously [33]. The baseline FNPO assessment was performed with a Voluson E8 Expert system (General Electric Systems®) and a 5–9 MHz transvaginal transducer, by counting all the follicles with a diameter of 2 to 9 mm, as described previously [28, 34]. The mean follicle count for the left and right ovaries was used in the statistical analysis.

### The COH protocol

All patients underwent a gonadotropin-releasing hormone (GnRH) agonist or antagonist protocol. In the agonist protocol, daily injections with triptorelin (0.1 mg) were initiated 1 week before the expected start of the following cycle. Desensitization was checked 12-15 days after initiation of the agonist protocol. When the E2 concentration was below 50 pg/mL and an ultrasound examination had confirmed the absence of functional ovarian cysts, COH was initiated with daily injections of recombinant FSH (rFSH) or human menopausal gonadotropin (HMG). In the antagonist protocol, women received daily injections of gonadotropins (rFSH or HMG) starting on day 2 of menstrual bleeding, followed by treatment with a GnRH antagonist (Ganirelix/Orgalutran®, MSD®, Courbevoie, France) starting on day 6 of the rFSH treatment. The starting dose of FSH ranged from 75 to 225 IU/d, depending on the body mass index (BMI), the antral follicle count, and the serum AMH level. The FSH dose was then set individually as a function of the E2 level and follicular growth during COH. Recombinant human chorionic gonadotropin (hCG, Ovitrelle®, Merck, Lyon, France) was administered when at least three follicles with a diameter > 17 mm were observed on ultrasound at the same time as a consistent rise in the serum E2 level. Oocytes were retrieved (using transvaginal ultrasound-guided needle aspiration) 36 h after the hCG injection.

### ICSI procedure and assessment of oocyte morphology

On the day of the ICSI, a semen sample was obtained by masturbation and collected in a sterile container. Sperm processing was performed with two discontinuous layers of PureSperm® (JCD, La Mulatière, France). After semen liquefaction, 1 to 2 mL of the ejaculates were layered in a sterile Falcon tube and centrifuged for 20 min at 300 x g. Following centrifugation, the supernatant was aspirated and discarded. Next, pellets containing selected spermatozoa were aspirated and transferred into new sterile Falcon tubes containing washing medium (Ferticult Hepes®, JCD, La Mulatière, France) and centrifuged at 300×g for 10 min. Pellet were re-suspended in 100 μL of Ferticult Hepes® medium and used for ICSI procedure. Sperm concentration and progressive sperm motility in the final preparation were assessed in the final preparation.

Two hours after the oocyte retrieval, the oocytes were stripped using chemical and mechanical methods. Each cumulus-oocyte complex was plunged for 20 s into a hyaluronidase solution (80 IU/mL, FertiPro®, France), following by successive aspirations and expulsions through a micropipette to remove the cumulus and the corona radiata cells. The oocytes’ meiotic maturity and morphology were evaluated by two independent operators at the time of the ICSI, using an inverted microscope (Leica DMIRB, Leica Microsystems®, Germany) and a magnification of × 400. A metaphase II oocyte was considered as morphologically normal when it exhibited a clear, homogenous cytoplasm with a uniform texture, with a smooth surface (with no vacuoles, smooth endoplasmic reticulum or granulations), a round-clear zona pellucida, with perivitelline space of normal size containing a single-non fragmented, round or ovoid, normal-sized first polar body.

The oocytes were screened for extracytoplasmic and intracytoplasmic abnormalities. The possible extracytoplasmic morphologic abnormalities were as follows: a fragmented or abnormal first polar body, an abnormal zona pellucida (thick, fine, or irregular), the presence of a large perivitelline space (PVS), the presence of material in the PVS, and an abnormally shaped oocyte. The possible intracytoplasmic morphological abnormalities included an abnormally granular cytoplasm (heterogeneous granulations or granulations concentrated in a central zone) and the presence of one or more vacuoles. In accordance with international guidelines, huge oocytes and/or oocytes containing inclusions of smooth endoplasmic reticulum were not injected [35]. Two oocyte morphology scores were determined: the average oocyte quality index (AOQI) [36] and the metaphase (M)II oocyte morphologic score (MOMS) [37]. The AOQI is calculated by first rating each oocyte for the presence (score: 1) or absence (score: 0) of each of the following morphologic abnormalities: granular cytoplasm, an abnormal zona pellucida, a fragmented or abnormal polar body, a large PVS, material in the PVS, an abnormal shape, and vacuoles. The AOQI then corresponds to the ratio between the total number of abnormalities counted and the total number of MII oocytes. The MOMS quantifies the severity of the morphologic characteristics in relation to the associated ICSI outcome; oocytes with a low MOMS rate have a greater implantation potential than those with a high MOMS rate (Table 2).

**Table 2:** Metaphase II oocyte morphological score (MOMS, from Rienzi et al., 2008 [37])

	Points
**Extracytoplasmic features**	
Abnormal first polar body	2.0
Large perivitelline space	1.4
**Cytoplasmic features**	
Granular cytoplasm	1.4
Centrally located granular area	2.7
Vacuoles	2.1

As reported previously, our evaluation of morphologic oocyte quality is highly reproducible (as judged by the level of inter-operator agreement) [36].

### Evaluation of embryos and ICSI cycles

Each MII oocyte was microinjected as part of a ICSI procedure described previously [38]. Fertilization (the presence of two distinct pronuclei) was scored 16-18 h after injection. Morphological embryo quality (i.e. the number of blastomeres, cell symmetry and fragmentation) was evaluated on day 2 or 3 according to the European Society of Human Reproduction and Embryology classification [35] A “top-quality embryo” was defined as the presence of four blastomeres on day 2 or eight blastomeres on day 3, symmetrical cleavage, and a fragmentation rate below 10% [35]. Top-quality embryos were scored as grade 1, and poor-quality embryos were scored as grade 3. Supernumerary top-quality embryos were frozen for subsequent transfer. Ultrasound-guided embryo transfer was performed 2 or 3 days after oocyte retrieval.

Luteal phase support (vaginal micronized progesterone 200 mg, 3 times a day) was indicated for all patients and was initiated on the evening of oocyte retrieval. The small proportion of freeze-all cycles (no fresh embryo transfers) was not taken into account in the comparisons of the ICSI outcomes.

### Outcomes

For the PCOS, PCOM-only and control groups, oocyte quality and morphology were assessed at the time of ICSI injection with regard to the oocyte maturity index (defined as the ratio between the number of MII oocytes and the total number of oocytes collected), the seven extracytoplasmic and intracytoplasmic abnormalities mentioned above, the AOQI, and the MOMS were performed.

After excluding cycles using frozen-thawed sperm, we evaluated the fertilization rate (FR), implantation rate (IR), clinical pregnancy rate (CPR), live birth rate (LBR) per transfer for day 2-3 embryos in fresh cycles, miscarriage rate (MR), and cumulative CPR. The IR was defined as the ratio between the number of gestational sacs with fetal heart activity and the number of embryos transferred. The live birth rate was defined as the number of live births per embryo transfer in fresh cycles. The cumulative CPR pregnancy rate was defined as the number of pregnancies with at least one gestational sac exhibiting fetal heart activity, including embryo transfers in fresh cycles and frozen-thawed cycles performed between 2006 and 2016.

### Statistical analysis

Quantitative variables were expressed as the mean ± standard deviation (for normally distributed datasets) or as the median [interquartile range (IQR)] otherwise. Normality of distributions was checked graphically and using Shapiro Wilk test. Categorical variables were expressed by frequencies and percentages. Comparisons of subject’s characteristics (at the first cycle) between the three PCOS phenotypes were performed with Chi-square test for current smoker women; and with analysis of variance for normally distributed quantitative variables or Kruskal-Wallis test otherwise. Post-hoc pairwise comparisons were performed in case of global significant difference and a Bonferroni correction was applied.

Groups were compared (i.e. PCOS vs PCOM-only vs controls; PCOS phenotypes A vs. C vs. D) using generalized estimating equation (GEE) models (to account for the correlation between repeated cycles in the same woman). A GEE model with binomial distribution and a logit link function was used for the binary dependent variables (agonist protocol, the number of normal oocytes and the number of each abnormality on all the oocytes, clinical pregnancy rate, live birth rate, miscarriage rate), and a GEE model with Poisson distribution and a logit link function was used for count outcomes (the total number of retrieved oocytes, the number of matured oocytes, number of day 2-3 embryos obtained, number of frozen embryos and the number of fresh transferred embryos, cumulative CPR per cycle); and for ratios (proportion of MII oocytes, fertilization rate, grade 1 embryos rate, grade 3 embryos rate, implantation rate, cumulative CPR per transfer) by considering the numerators as dependent variable and the denominators as an offset variable. A linear mixed model with the patient as a random effect was used for comparisons of quantitative dependent variables (age of women, number of stimulation days, total dose of FSH, Estradiol on hCG day, AOQI score per cycle, average MOMS score per cycle). Comparisons of cycle outcomes were further adjusted for well-established, pre-specified confounding factors (namely the woman’s age, BMI, smoking status, COH, and total dose of FSH). When analyzing the CPR for fresh embryos transfers, the number of fresh embryos transferred was also considered as a confounding factor. For the two cumulative CPRs (per cycle, and per total number of transfers), the number of total embryos transferred was considered as additional confounding factor. All statistical analyses were performed with SAS software (version 9.4, SAS Institute Inc., Cary, NC, USA). All tests were two-tailed, and the threshold for statistical significance was set to *p* < 0.05.

## Results

### The study population

A total of 109 women (aged between 22 and 37) were diagnosed with PCOS and had undergone a total of 189 ICSI cycles.

The clinical and endocrine characteristics of the phenotype groups are summarized in Table 3. There were no statistically significant differences between the three phenotype groups with regard to the woman’s age at the time of the first ICSI attempt, the BMI, the male partner’s age, or the FSH levels. The serum AMH level was significantly higher for phenotype A (74.8 ± 37.4) than for phenotypes C (47.3 ± 17.1) and D (56.2 ± 19.9) (*p* < 0.001). Androstenedione levels were significantly higher for phenotypes A and C than for phenotype D. The only significant difference in the serum testosterone concentration concerned phenotypes A and D (respectively, 0.4 ± 0.2 vs 0.3 ± 0.1; *p* = 0.004). The characteristics of the COH and outcomes for respectively 77, 57 and 55 cycles in the phenotype A, C and D groups are summarized in Table 4. There were no significant intergroup differences in the proportion of agonist protocols, the total dose of gonadotropin, and the total numbers of retrieved and MII oocytes.

**Table 3:** Clinical and endocrine characteristics, by PCOS phenotype

*PCOS B (n = 1) was excluded*	PCOS A ***N*** = 41	PCOS C ***N*** = 31	PCOS D ***N*** = 37	***p*** value
Woman’s age at first ICSI cycle (y)	29.2 ± 3.2	28.6 ± 4.1	29.1 ± 3.4	0.77
Man’s age (y)	32.9 ± 4,5	32.0 ± 7,2	32.6 ± 4,5	0.80
Current smoking, n (%)	9 (22.0)	14 (45.2) ^c^	5 (13.5) ^b^	0.009
BMI (kg/m^2^)	27.7 ± 6.7	26.4 ± 6.0	26.3 ± 5.0	0.48
Waist circumference (cm)	86.6 ± 16.3	84.0 ± 14.8	84.6 ± 12.8	0.74
Testosterone (ng/mL)	0.4 ± 0.2^c^	0.3 ± 0.2	0.3 ± 0.1^a^	0.004
Delta-4-androstenedione (ng/mL)	2.3 ± 0.7^c^	2.1 ± 0.7^c^	1.3 ± 0.4^ab^	< 0.001
LH (IU/L)	5.6 ± 2.6 ^bc^	4.1 ± 1.9^a^	4.2 ± 2.0^a^	0.006
FSH (IU/L)	4.8 ± 1.1	5.2 ± 1.4	4.7 ± 1.0	0.23
LH/FSH ratio (IU/L)	1.1 [0.9-1.4] ^bc^	0.8 [0.6-1.0] ^a^	0.8 [0.7-1.2] ^a^	< 0.001
AMH (pmol/L)	74.8 ± 37.4 ^bc^	47.3 ± 17.1^a^	56.2 ± 19.9^a^	< 0.001
FNPO (n)	26.8 ± 10.1	21.7 ± 8.2	23.1 ± 8.0	0.042

Values are quoted as the mean ± standard deviation, the mean [IQR], or n (%)

^a^ *p* < 0.05 vs. PCOS A after Bonferroni correction

^b^ *p* < 0.05 vs. PCOS C after Bonferroni correction

^c^ *p* < 0.05 vs. PCOS D after Bonferroni correction

Abbreviations: *y* years, *BMI* body mass index, *LH* luteinizing hormone, *FSH* follicle-stimulating hormone, *IQR* interquartile range, *AMH* anti-Müllerian hormone, *FNPO* follicle number per ovary

**Table 4:** Clinical and laboratory characteristics in ICSI cycles, by PCOS phenotype

*PCOS B (n = 1) was excluded*	PCOS A ***N*** = 41	PCOS C ***N*** = 31	PCOS D ***N*** = 37	***p*** value	Adjusted p
Number of ICSI cycles	77	57	55	–	–
Woman’s age during the ICSI cycle	29.6 ± 3,3	29.0 ± 4.3	29.7 ± 3.2	0.48	–
Protocol:				0.73	–
Agonist, n (%)	38 (49.4)	29 (50.9)	24 (43.6)	–	–
Antagonist, n (%)	39 (50.6)	28 (49.1)	31 (56.4)	–	–
Number of days of stimulation	12.9 ± 2.5	12.1 ± 2.5	12.1 ± 1.9	0.15	–
Total dose of FSH, IU	1812 ± 872	2016 ± 1076	1741 ± 700	0.41	–
Estradiol on hCG day, pg/mL	2666 ± 1454	2363 ± 1387	2382 ± 1206	0.42	–
Mean number of oocytes retrieved (N)	12.2 ± 6.0	12.1 ± 5.2	11.8 ± 6.2	0.92	0.92
Mean number of MII oocytes (n)	7.8 ± 4.6	8.1 ± 3.6	7.9 ± 4.8	0.91	0.98
Proportion of MII oocytes, n/N (%)	602/939 (64.1)	462/692 (66.8)	432/648 (66.7)	0.85	0.94

Values are quoted as the mean ± standard deviation or n (%)

The *p* value was adjusted for the woman’s age, BMI and current smoking status, the type of COH protocol, and the total dose of FSH

Abbreviations: *FSH* follicle-stimulating hormone, *MII* metaphase II

The clinical and endocrine characteristics of the PCOS, PCOM-only and control groups are summarized in supplemental Table 1. The BMI was significantly higher in the PCOS group than in the control group (*p* = 0.025). The waist circumference was significantly higher in the PCOS group than in the control group (85.0 ± 14.7 vs 78.5 ± 12.3, *p* = 0.012). The serum testosterone level and the serum delta-4 androstenedione level were significantly higher (*p* < 0.001 and < 0.001, respectively) in the PCOS group than in the PCOM-only and control groups. Furthermore, the LH/FSH ratio was significantly higher in the PCOS group than in the two other groups. The antral follicle count and serum AMH levels were significantly higher (i) in the PCOS group than in the PCOM-only and control groups, and (ii) in the PCOM-only group than in the control group (Supplemental Table 1). There were no significant intergroup differences in the woman’s age, the duration of stimulation, and the serum estradiol level on the trigger day. The numbers of retrieved oocytes and MII oocytes were significantly higher in the PCOS and PCOM-only groups per cycle (respectively: 12.0 ± 5.7; 7.9 ± 4.3) and PCOM-only groups (respectively: 12.4 ± 6.2; 7.9 ± 4.2) than in control group (respectively: 9.5 ± 4.7; 6.2 ± 3.9). However, no difference was found in the mature oocyte rate between the three groups (supplemental Table 2).

### Oocyte morphology comparison

A total of 3312 oocytes were assessed morphologically: 1523 oocytes in the PCOS group; 1059 in the PCOM-only group; 730 in the control group.

After adjustment for the woman’s age, BMI and smoking status and the total dose of FSH administered, a detailed analysis of each morphologic abnormality did not reveal any significant differences between the three PCOS phenotypes (Table 5). The percentages of normal oocytes were similar for PCOS phenotypes A, C and D (percentages (95%CI): 35.2% (31.5 to 39.1%), 25.8% (21.9 to 29.9%) and 34.0% (29.7 to 38.6%), adjusted *p* = 0.13). Similarly, there were no intergroup differences in oocyte morphologic quality (as expressed by the AOQI (means (95% CI)) for A, C and D respectively: 0.76 (0.68 to 0.85), 0.85 (0.72 to 0.98), 0.75 (0.64 to 0.86), adjusted *p* = 0.40) and the MOMS for A, C and D respectively: 1.31 (1.04-1.46), 1.30 (1.10-1.50), 1.27 (1.08-1.46), adjusted *p* = 0.83)).

**Table 5:** Oocyte morphologic quality, by PCOS phenotype

	PCOS A ***N*** = 41	PCOS C ***N*** = 31	PCOS D ***N*** = 37	***p*** value	Adjusted p
Number of MII oocytes	602	462	432	–	–
Normal oocytes, n (%)	212 (35.2 [31.5 to 39.1])	119 (25.8 [21.9 to 29.9])	147 (34.0 [29.7 to 38.6])	0.031	0.13
Fragmented or abnormal FPB, n (%)	299 (49.7 [45.7 to 53.7])	245 (53.0 [48.5 to 57.5])	241 (55.8 [51.1 to 60.4])	0.16	0.15
Abnormal zona pellucida, n (%)	8 (1.3 [0.7 to 2.6])	6 (1.3 [0.6 to 2.9])	3 (0.7 [0.2 to 2.1])	0.60	0.75
Large perivitelline space, n (%)	49 (8.1 [6.2 to 10.6])	56 (12.1 [9.4 to 15.4])	29 (6.7 [4.7 to 9.5])	0.36	0.34
Material in perivitelline space, n (%)	68 (11.3 [9.0 to 14.1])	52 (11.3 [8.9 to 14.5])	41 (9.5 [7.1 to 12.6])	0.89	0.80
Abnormal shape of oocyte, n (%)	19 (3.2 [2.0 to 4.9])	14 (3.0 [1.8 to 5.1])	9 (2.1 [1.1 to 4.0])	0.41	0.39
Granular cytoplasm, n (%)	21 (3.5 [2.3 to 5.3])	16 (3.5 [2.1 to 5.6])	16 (3.7 [2.3 to 6.0])	0.92	0.81
Intracytoplasmic vacuoles, n (%)	12 (2.0 [1.1 to 3.5])	15 (3.3 [2.0 to 5.3])	3 (0.7 [0.2 to 2.1])	0.049	0.053
AOQI, mean ± SD^a^	0.76 [0.68 to 0.85]	0.85 [0.72 to 0.98]	0.75 [0.64 to 0.86]	0.29	0.40
average MOMS per cycle, mean ± SD^a^	1.31 [1.04 to 1.46]	1.30 [1.10 to 1.50]	1.27 [1.08 to 1.46]	0.80	0.83

Values are quoted as the mean [95%CI] or n (% [95%CI]). The *p* value was adjusted for the woman’s age, BMI and current smoking status, the type of COH protocol, and the total dose of FSH

Abbreviations: *MII* metaphase II, *FPB* first polar body, *ZP* zona pellucida, *AOQI* average oocyte quality index, *MOMS* metaphase II oocyte morphologic score

^a^The AOQI score considers all the oocyte morphologic abnormalities per cycle (Sigala et al., 2015 [36]), while the MOMS takes account of the morphologic abnormalities per oocyte. However, the MOMS was averaged by cycle here, in order to compare the groups (Rienzi et al., 2008 [37])

Lastly, there were no differences between the PCOS, PCOM-only and control groups in the normal oocyte rate (adjusted percentages (95%CI): 31.8% (29.5 to 34.2%), 30.6% (27.9 to 33.4%), 27.8% (24.7 to 31.2%) respectively, adjusted *p* = 0.90), the AOQI or the MOMS (Supplemental Table 3).

### ICSI outcomes

After the exclusion of ICSI cycles requiring frozen-thawed sperm, the ICSI outcomes are shown in Table 6. There was no significant phenotype-related difference in the fertilization rate (percentages (95%CI) for A, C and D respectively: 61.8% (55.4 to 68.8%), 59.1% (52.0 to 67.2%), 59.2% (51.7 to 67.9%), adjusted *p* = 0.93) or the mean number of day 2-3 embryos obtained (mean (95% CI)) for A, C and D respectively: 4.6 (4.1-5.1), 4.6 (4.1-5.3), 4.8 (4.2-5.5), *p* = 0.84). The percentages of good quality (grade 1) embryos (percentage (95%CI) for A, C and D respectively: 49.8% (42.7 to 58.2%), 50.9% (42.3 to 61.2%), 51.7% (42.7 to 62.7%), adjusted *p* = 0.86) and poor quality (grade 3) embryos (percentage (95%CI) for A, C and D respectively: 40.8% (34.3 to 48.4%), 40.1% (32.5 to 49.4%), 37.3% (29.7 to 46.8%), adjusted *p* = 0.58) were also similar in the three phenotype groups. After adjustment for confounding factors we did not detect any intergroup differences in the IR, CPR, LBR, MR, and cumulative CPR. With regard to ICSI outcomes, only the number of D2-D3 embryos and the number of frozen embryos were significantly higher in the PCOS group than in the PCOM and control groups (Supplemental Table 4).

**Table 6:** Embryo morphologic quality and ICSI outcomes, by PCOS phenotype

	PCOS A	PCOS C	PCOS D	***p*** value	Adjusted p
Number of ICSI cycles	70	48	42	–	–
Fertilization rate, n/n_MII ovo_ (%)	331/536 (61.8 [55.4 to 68.8])	233/394 (59.1 [52.0 to 67.2])	205/346 (59.2 [51.7 to 67.9])	0.93	0.93
Number of day 2-3 embryos	4.6 [4.1 to 5.1]	4.6 [4.1 to 5.3]	4.8 [4.2 to 5.5]	0.92	0.84
Grade 1 embryos (%)^ǂ^	159/319 (49.8 [42.7 to 58.2])	113/222 (50.9 [42.3 to 61.2])	104/201 (51.7 [42.7 to 62.7])	0.93	0.86
Grade 3 embryos (%)^ǂ^	130/319 (40.8 [34.3 to 48.4])	89/222 (40.1 [32.6 to 46.8])	75/201 (37.3 [29.8 to 46.8])	0.76	0.58
Number of frozen embryos (n) ^ǂ^	1.7 [1.4 to 2.0]	1.5 [1.2 to 1.9]	2.0 [1.6 to 2.5]	0.59	0.57
Fresh transferred embryos (n)^ǂ^	1.4 [1.1 to 1.7]	1.6 [1.3 to 2.0]	1.4 [1.1 to 1.8]	0.42	0.39
Implantation rate, n/n_embryos transferred_ (%)*	28/89 (31.5 [21.7 to 45.6])	21/73 (28.8 [18.8 to 44.1])	18/56 (32.1 [20.3 to 51.0])	0.79	0.82
CPR, n/n_transfers with fresh embryos_ (%)*	26/53 (49.1 [36.0 to 62.3])	20/39 (51.3 [36.0 to 66.4])	15/33 (45.5 [29.6 to 62.3])	0.88	0.71^ƒ^
Live birth rate, n/n_transfers with fresh embryos_ (%)*	19/53 (35.8 [24.2 to 49.5])	16/39 (41.0 [26.9 to 56.8])	13/33 (39.4 [24.4 to 56.6])	0.92	0.44
Miscarriage rate, n/n_clinical pregnancy_ (%)*	5/26 (19.2 [8.2 to 38.7])	3/20 (15.0 [4.9 to 37.6])	2/15 (13.3 [3.4 to 40.5])	0.89	NA
Cumulative CPR, n/n_total transfers_ (%)	33/94 (35.1 [25.0 to 49.4])	26/65 (40.0 [27.2 to 58.8])	29/68 (42.6 [28.4 to 59.6])	0.51	0.43^#^
Cumulative CPR, n/n_cycles_ (%)	33/70 (47.1 [33.5 to 66.3])	26/48 (54.2 [36.9 to 79.6])	29/42 (69.0 [48.0 to 99.4])	0.15	0.18^#^

Values are quoted as the mean [95% CI] or no./total no. (rate [95%CI] in %). The *p* value was adjusted for the woman’s age, BMI and current smoking status, the type of COH protocol, and the total dose of rFSH

ƒ additionally adjusted for the number of fresh embryos transferred

# additionally adjusted for the total number of embryos transferred

Abbreviations: *CPR* clinical pregnancy rate, *NA* not applicable, due to low frequencies

ǂ cycles with no embryos obtained on day 2-3 were excluded

* cycles with embryo transfer on day 5-6 and cycles with a “freeze-all” strategy were excluded

## Discussion

Although a few published studies have investigated reproductive IVF/ICSI outcomes among women with PCOS, oocyte morphologic quality for the various PCOS phenotypes has not previously been described. Indeed, previous research by our group found that oocyte quality was similar in a PCOM group and a control women; however, the study’s PCOM group contained a mixture of PCOM-only women and PCOS women [36]. It is well established that PCOM is a distinct phenotype and is not the same as PCOS [31]. We therefore decided to investigate oocyte morphology among the different PCOS phenotypes. To the best of our knowledge, the present study is the first to have addressed this topic. Our study population was homogenous and met strict inclusion criteria - including ICSI for male infertility only. All cases with potential female infertility other than PCOS were excluded.

Importantly, we did not detect any significant differences in oocyte morphologic quality or embryo quality between the PCOS phenotypes A, C and D. This unexpected observation challenges a number of preconceived ideas. Firstly, some researchers have reported that relative to healthy women in IVF and ICSI programs, phenotypes A and B (considered to the most severe, with an increased risk of comorbidities such as hyperinsulinism and metabolic disorder) are associated with a lower pregnancy rate [18] and a greater risk of adverse outcomes in pregnancy [16]. It has been suggested that these poor outcomes are related to low oocyte quality or competence. Secondly, the cardinal features of PCOS (i.e. OA, HA, and PCOM) are suspected to have an adverse impact (independently or combined) on oocyte quality [15].Indeed, androgens are involved in folliculogenesis, and a hyperandrogenic environment leads to abnormal folliculogenesis, prematurely activated follicles, mitochondrial abnormalities, and failure of meiosis progression to MII. Furthermore, HA is known to induce premature luteinization of the granulosa cells, which prevents them from progressing to physiological atresia. It has been shown that OA is associated with an alteration in the insulin growth factor (IGF) pathway, which is involved in embryonic development and blastocyst formation [12]. However, the true extent of the IGF pathway’s involvement in the pathogenesis of PCOS is still unknown. Hence, the impact of oligo-anovulation on oocyte quality and reproductive outcomes (with the exception of the pregnancy rate per cycle) has yet to be determined [15]. Likewise, the impact of HA on oocyte quality is subject to debate. Some researchers have highlighted a negative effect of androgens on oocyte maturity in animal models [39], and others have demonstrated the androgens’ fundamental role in early folliculogenesis and the pre-ovulatory follicular stages [40]. The elevated androgen levels in PCOS might lead to excess AMH secretion, which in turn is involved in so-called “follicular arrest” in the ovaries [34, 41]. In contrast, Gaddas and colleagues (2016) did not find any negative impact of biochemical HA on conventional IVF or ICSI outcomes in women with PCOS [17]. Furthermore, a study by Palomba and colleagues (2010) did not show any significant effects of clinically defined HA and PCOM (PCOS phenotype C) [16], which is in line with our present results. Lastly, our comparison of oocyte quality in PCOS, PCOM-only and controls gave much the same results as the only two other studies to have evaluated morphologic abnormalities of the oocytes [10, 36].

Our present results confirmed the above-mentioned observation for clinical and endocrine features in PCOS phenotype groups. Firstly, we did not find any differences in the woman’s BMI between the three A, C and D phenotypes - as also reported in a recent case-control study [42]. Secondly, the PCOS phenotype A population exhibited the highest AMH levels, which is in line with previous reports [18, 19, 42, 43]. Furthermore, the severity of OA appeared to be correlated with an elevated serum AMH level [44, 45]. We discovered that serum delta-4-androstenedione levels were higher for PCOS phenotype A than for phenotype D, and that LH and AMH levels were higher for phenotype A than for phenotypes C and D. These results are also in line with another recent report [19].

It is acknowledged that oocyte morphology is an easily assessed, non-invasive marker of oocyte quality. Some oocyte abnormalities (i.e. a large perivitelline space, the presence of cytoplasmic vacuoles, or an abnormal shape) are known to be associated with poor reproductive outcomes [33, 37, 40, 41, 46]. In contrast, some researchers did not find any differences in these morphological parameters [34, 47, 48]. Likewise, our study did not evidence an adverse relationship between individual oocyte morphologic abnormalities and the ICSI outcomes. Consequently, the oocyte morphology’s predictive value is still subject to debate [49–52]. We therefore assessed oocyte quality with regard to not only the extra- and intra-cytoplasmic abnormalities described above but also two scoring systems. Each oocyte abnormality is considered separately in the AOQI, [36], whereas the presence of specific oocyte features are computed in the MOMS. In the present study, we did not detect any differences between the PCOS phenotypes A, C and D with regard to the AOQI or the MOMS.

In line with previous reports of similar embryo quality in PCOS and non-PCOS patients [8, 10, 53, 54], we did not observed interphenotype differences in these variables or, indeed, in reproductive outcomes. This contrast with recent reports in which PCOS A and B were associated with a lower clinical pregnancy rate [18] and PCOS phenotypes with HA were associated with a lower cumulative live birth rates, when compared with normo-androgenic counterparts [55]. This discrepancy might be due to our strict inclusion criteria and thus our small study population. Due to our relatively small study sample size, we cannot exclude that several associations were overlooked due to a lack of adequate statistical power. For this reason, our results regarding reproductive outcomes, should be interpreted with caution and furthers studies are warranted to confirm our findings.

Our study had a number of limitations. Firstly, we cannot draw any conclusions with regard to the PCOS phenotype B because we used the serum AMH level as a surrogate marker of PCOM. In fact, our patients with PCOS patients were diagnosed according to the revised Rotterdam criteria [3, 25, 33]. Secondly, we used a serum AMH threshold concentration > 35 pmol/l for the PCOM group in our study [28, 29] This threshold was established with an assay kit that is no longer commercially available [56]. For consistency, the inclusion period for the PCOS, PCOM-only and control groups was restricted to the time when we used the AMH assay; this also explains why our study population was small. Some researchers consider that the serum AMH level is a more reproducible, more sensitive variable than the FNPO [16, 28, 29, 44, 57, 58]. The FNPO threshold of 12 in the 2003 Rotterdam criteria is outdated and leads to overestimation of the prevalence of PCOM in the general population [32, 59]. Consequently, we decided to use the FNPO threshold of 19 reported in a cluster analysis [28]. This value is also close to that given in international evidence-based guidelines for the assessment and management of PCOS from 2018 [4]. An AMH assay and pelvic ultrasound should be used together to determine the presence of PCOM and thus correct a false negative for one or the other. Hence, their combined use, causes the phenotype B to almost completely disappear [29]. Nevertheless, we would like to point out that the results in terms of pregnancy rates of ICSI cycles are presented for information purposes only; our aim was to focus on oocyte morphology as a possible marker of oocyte quality in women with PCOS. Therefore, it is difficult to compare the pregnancy rates results with previous studies, since our classification into A, B, C and D phenotypes is also based on the result of AMH levels [29], unlike those other studies [18, 55]. Indeed, in our experience, a high AMH level with a specific threshold adapted to the assay technique and determined in cluster analysis can be considered as a biological equivalent of PCOM as we previously published [28, 44, 56, 60]. Moreover, in these studies, several confounding factors that could affect the results were not taken into account in the statistical analysis, such as smoking status or additional causes of women infertility (endometriosis).

Secondly, our study’s retrospective, single-center design was associated with inherent bias. Nonetheless, the single-center design enabled us to generate novel data in a homogeneous patient population. All the women received standardized care, and the proportion of missing data was very low - constituting key study strengths. Furthermore, we used very strict inclusion and non-inclusion criteria, notably with regard to any differential diagnoses; this also explains why the study population was relatively small. Furthermore, we used Poisson and logistic regressions to assess the effect of potential confounding factors. One limitation of this study was represented by the technical improvement of ultrasound over the past 10 years which can impact the antral follicle assessment, in particular regarding small follicles. Nevertheless, our FNPO values were all obtained with transvaginal ultrasound using a 5–9 mHz probe, based on a routinely standardized protocol in order to minimize the impact of technology improvement on follicle counting.

Additionally, the type of gonadotropin used in ovarian stimulation in patients undergoing IVF/ICSI was not taken into account in the interpretation of oocyte morphology in PCOS women. Indeed, most of the data are rather in favor of an equal efficiency of the different types of gonadotropins during a COH protocol. The recent ESHRE recommendations concerning ovarian stimulation in IVF states that “The use of recombinant FSH (rFSH) and human menopausal gonadotropin (hMG) for ovarian stimulation is equally recommended” [61, 62]. However, some studies have assessed the impact of gonadotropin used for COH protocol in the oocyte and/or the embryo quality [63, 64]. The study by Ziebe et al. [63] has assessed the effects of gonadotropin type on early embryonic morphology up to day 3 of development, which appeared to be somewhat better with hMG, but it did not include women with PCOS. Indeed, only one study has assessed the impact of gonadotropin type used for COH protocol in women with PCOS [64], showing a significantly lower estradiol peak (E2), fewer intermediate-sized follicles, lower number of oocytes retrieved and MII oocytes in hMG-group in comparison with recombinant FSH-group. However, there were no significant differences in the number of fertilized oocytes, fertilization rates, top embryo quality, nor in the pregnancy rates between the two groups. Additionally, the authors did not assess the oocyte morphology.

Finally, we did not investigate whether the metabolic profile (in particular insulin resistance and hyperinsulinemia) in PCOS women could impact oocyte morphology in our study. Indeed, some authors have demonstrated in a mice model that insulin resistance impaired both oocyte quality and embryo development [65]. By consequent, the comparison of detailed metabolic profile in PCOS women in addition to the PCOS phenotype (according Rotterdam) could be of interest in the evaluation of oocyte quality and ICSI outcomes in futures prospective studies.

In summary, our results showed that PCOS A, C and D phenotypes did not differ significantly with regard to oocyte morphologic quality and thus suggest that no single PCOS phenotype is associated with poor quality. Our results also suggest that the phenotype A (considered to be the most severe phenotype) is not associated with especially poor reproductive outcomes. Further studies are required to confirm these findings and characterize the underlying mechanisms.

## Supplementary Information

**Additional file 1.**

## Data Availability

The data sets used and/or analyzed during the current study are available from the database of the Lille University Medical IVF center’s on reasonable request.

## Abbreviations

17-OHP: 17-hydroxyprogesterone
AMH: Anti-Müllerian hormone
AOQI: Average oocyte quality index
BMI: Body mass index
COH: Controlled ovarian hyperstimulation
CPR: Clinical pregnancy rate
E2: Estradiol
FNPO: Follicle number per ovary
FR: Fertilization rate
GEE: Generalized estimating equation
GnRH: Gonadotropin-releasing hormone
HA: Hyperandrogenism
hCG: Human chorionic gonadotropin
ICSI: Intracytoplasmic sperm injection
IQR: Interquartile range
IR: Implantation rate
IVF: In vitro fertilization
LBR: Live birth rate
MOMS: Metaphase (M)II oocyte morphologic score
MR: Miscarriage rate
OA: Oligo-ovulation/anovulation
PCOM: Polycystic ovarian morphology
PCOS: Polycystic ovary syndrome
PVS: Perivitelline space
TT: Total testosterone
