# Supplementary material for: Polycystic ovary syndrome phenotype does not have impact on oocyte morphology
Source: Reprod Biol Endocrinol. 2022 Jan 5;20:7. doi: 10.1186/s12958-021-00874-2 (PMC8729101; doi:10.1186/s12958-021-00874-2)
Supplement: Supplementary file 1 — Additional file 1. [file 12958_2021_874_MOESM1_ESM.docx]

**Tables**

**Supplemental Table 1:** Clinical and endocrine characteristics

|  | **PCOS**  **N=110** | **PCOM-only**  **N=65** | **Controls**  **N=58** | **p value** |
| --- | --- | --- | --- | --- |
| Woman’s age at first ICSI cycle (y) | 29.0 ± 3.5 | 29.6 ± 3.1 | 29.2 ± 3.8 | 0.54 |
| Man’s age (y) | 32.5 ± 5.3 | 32.2 ± 3.9 | 33.7 ± 7.4 | 0.39 |
| Current smoker, n (%) | 28 (25.5) | 11 (16.9) | 16 (27.6) | 0.31 |
| BMI (kg/m^2^) | 26.8 ± 5.9^c^ | 25.1 ± 4.9 | 24.6 ± 5.6^a^ | 0.025 |
| Waist circumference (cm) | 85.0 ± 14.7^c^ | 81.7 ± 12.0 | 78.5 ± 12.3^a^ | 0.012 |
| Testosterone (ng/mL) | 0.3 ± 0.2^bc^ | 0.2 ± 0.1^a^ | 0.2 ± 0.1^a^ | <0.001 |
| Delta-4-androstenedione (ng/mL) | 1.9 ± 0.7^bc^ | 1.3 ± 0.4^a^ | 1.1 ± 0.3^a^ | <0.001 |
| LH (IU/L) | 4.7 ± 2.3^bc^ | 3.7 ± 1.5^a^ | 3.5 ± 1.3^a^ | <0.001 |
| FSH (IU/L) | 4.9 ± 1.2^c^ | 5.0 ± 1.0^c^ | 5.7 ± 1.4^ab^ | <0.001 |
| LH/FSH ratio (IU/L) median [IQR] | 0.85 [0.68-1.17]^bc^ | 0.7 [0.60-0.87]^ac^ | 0.59 [0.47-0.74]^ab^ | <0.001 |
| AMH (pmol/L) | 60.3 ± 29.5^bc^ | 46.2 ± 16.8^ac^ | 22.1 ± 8.3^ab^ | <0.001 |
| FNPO | 23.9 ± 9.2^bc^ | 19.9 ± 5.8^ac^ | 11.0 ± 2.6^ab^ | <0.001 |

Values are quoted as the mean ± standard deviation, the mean [IQR], or n (%).

^a^ p<0.05 vs. PCOS after Bonferroni correction

^b^ p<0.05 vs. PCOM-only after Bonferroni correction

^c^ p<0.05 vs. controls after Bonferroni correction

Abbreviations: y = years, BMI = body mass index, LH = luteinizing hormone, FSH = follicle-stimulating hormone, IQR = interquartile range, AMH = anti-Müllerian hormone, FNPO = follicle number per ovary

**Supplemental Table 2:** Clinical and laboratory characteristics in ICSI cycles

|  | **PCOS**  **N=110** | **PCOM-only**  **N=65** | **Controls**  **N=58** | **p value** | **Adjusted**  **p** |
| --- | --- | --- | --- | --- | --- |
| Number of ICSI cycles | 192 | 134 | 118 | - | - |
| Woman’s age during the ICSI cycle | 29.4 ± 3.6 | 30.2 ± 3.3 | 30.2 ± 4.0 | 0.094 | - |
| Protocol: |  |  |  | 0.003 | - |
| Agonist, n (%) | 91 (47.4)^c^ | 74 (55.2) | 80 (67.8)^a^ | - | - |
| Antagonist, n (%) | 101 (52.6) ^c^ | 60 (44.8) | 38 (32.2) ^a^ | - | - |
| Number of days of stimulation | 12.4 ± 2.4 | 12.1 ± 3.1 | 11.7 ± 2.0 | 0.13 | - |
| Total dose of FSH, IU | 1857 ±892^c^ | 1696 ± 633^c^ | 2559 ± 1238^ab^ | <0.001 | - |
| Estradiol on hCG day, pg/mL | 2482 ± 1359 | 2520 ± 1203 | 2466 ± 1022 | 0.97 | - |
| Mean number of oocytes retrieved (N) | 12.0 ± 5.7^c^ | 12.4 ± 6.2^c^ | 9.5 ± 4.7^ab^ | 0.001 | <0.001 |
| Mean number of MII oocytes (n) | 7.9 ± 4.3^c^ | 7.9 ± 4.2^c^ | 6.2 ± 3.9^ab^ | 0.014 | 0.006 |
| Proportion of MII oocytes, n/N (%) | 1523/2310 (65.9) | 1059/1666 (63.6) | 730/1122 (65.1) | 0.77 | 0.57 |

Values are quoted as the mean ± standard deviation or n (%).

^a^ p<0.05 vs. PCOS after Bonferroni correction

^b^ p<0.05 vs. PCOM-only after Bonferroni correction

^c^ p<0.05 vs. controls after Bonferroni correction

The p value was adjusted for the woman’s age, BMI and current smoking status, the type of COH protocol, and the total dose of FSH.

Abbreviations: FSH = follicle-stimulating hormone, MII = metaphase II

**Supplemental Table 3:** Oocyte morphologic quality

|  | **PCOS**  **N=110** | **PCOM-only**  **N=65** | **Controls**  **N=58** | **p value** | **Adjusted p** |
| --- | --- | --- | --- | --- | --- |
| Number of MII oocytes | 1523 | 1059 | 730 | - | - |
| Normal oocytes, n (%) | 484  (31.8 [29.5 to 34.2]) | 324  (30.6 [27.9 to 33.4]) | 203  (27.8 [24.7 to 31.2]) | 0.33 | 0.90 |
| Fragmented or abnormal FPB, n (%) | 792  (52.0 [49.5 to 54.5]) | 540  (51.0 [48.0 to 54.0]) | 386  (52.9 [49.3 to 56.5]) | 0.88 | 0.86 |
| Abnormal zona pellucida, n (%) | 17  (1.1 [0.7 to 1.8]) | 19  (1.8 [1.1 to 2.8]) | 20  (2.7 [1.8 to 4.2]) | 0.72 | 0.88 |
| Large perivitelline space, n (%) | 134  (8.8 [7.4 to 10.3]) | 95  (9.0 [7.4 to 10.8]) | 70  (9.6 [7.7 to 11.9]) | 0.75 | 0.86 |
| Material in perivitelline space, n(%) | 163  (10.7 [9.2 to 12.4]) | 124  (11.7 [9.9 to 13.8]) | 92  (12.6 [10.4 to 15.2]) | 0.35 | 0.30 |
| Abnormal shape of oocyte, n (%) | 42  (2.8 [2.0 to 3.7]) | 32  (3.0 [2.1 to 4.2]) | 15  (2.1 [1.2 to 3.4]) | 0.50 | 0.57 |
| Granular cytoplasm, n (%) | 70  (4.6 [3.7 to 5.8]) | 81  (7.7 [6.2 to 9.4]) | 49  (6.7 [5.1 to 8.8]) | 0.18 | 0.52 |
| Intracytoplasmic vacuoles, n (%) | 32  (2.1 [1.5 to 3.0]) | 52  (4.9 [3.8 to 6.4]) | 14  (1.9 [1.1 to 3.2]) | 0.58 | 0.42 |
| AOQI* | 0.79 [0.73 to 0.85] | 0.90 [0.81 to 0.99] | 0.82 [0.73 to 0.91] | 0.26 | 0.42 |
| average MOMS per cycle* | 1.30 [1.17 to 1.44] | 1.44 [1.25 to 1.63] | 1.57 [1.29 to 1.86] | 0.19 | 0.36 |

Values are quoted as the mean [95%CI] or n (% [95%CI]). The p value was adjusted for the woman’s age, BMI and current smoking status, the type of COH protocol, and the total dose of FSH.

Abbreviations: MII = metaphase II, FPB = first polar body, ZP = zona pellucida, AOQI = average oocyte quality index, MOMS = metaphase II oocyte morphologic score

*The AOQI score considers all the oocyte morphologic abnormalities per cycle (Sigala et al., 2015), while the MOMS takes account of the morphologic abnormalities per oocyte. However, the MOMS was averaged by cycle here, in order to compare the groups (Rienzi et al., 2008).

**Supplemental Table 4:** Embryo morphologic quality and ICSI outcomes

|  | **PCOS** | **PCOM-only** | **Controls** | **p value** | **Adjusted p** |
| --- | --- | --- | --- | --- | --- |
| Number of ICSI cycles | 163 | 92 | 94 | - | - |
| Fertilization rate, n/n_MII ovo_ (%) | 790/1303  (60.6 [56.5 to 65.0]) | 466/745  (62.6 [57.1 to 68.5]) | 352/629  (56.0 [50.4 to 62.1]) | 0.15 | 0.069 |
| Number of day 2-3 embryos | 4.7 [4.4 to 5.0]^c^ | 4.9 [4.5 to 5.4]^c^ | 3.5 [3.1 to 3.9]^ab^ | **0.004** | **<0.001** |
| Grade 1 embryos (%)^ǂ^ | 386/763  (50.6 [45.8 to 55.9]) | 197/451  (43.7 [38.0 to 50.2]) | 160/327  (48.9 [41.9 to 57.1]) | 0.74 | 0.67 |
| Grade 3 embryos (%)^ǂ^ | 299/763  (39.2 [35.0 to 43.9]) | 213/451  (47.2 [41.3 to 54.0]) | 145/327  (44.3 [37.7 to 51.2]) | 0.60 | 0.69 |
| Number of frozen embryos (n) ^ǂ^ | 1.7 [1.5 to 1.9]^c^ | 1.4 [1.2 to 1.7] | 1.0 [0.8 to 1.2]^a^ | **0.023** | **0.025** |
| Fresh transferred embryos (n)^ǂ^ | 1.5 [1.3 to 1.7] | 1.6 [1.4 to 1.9] | 1.6 [1.3 to 1.9] | 0.29 | 0.29 |
| Implantation rate, n/n_embryos transferred_ (%)* | 68/224  (30.4 [24.0 to 38.5]) | 31/142  (21.8 [15.4 to 31.0]) | 38/133  (28.6 [20.8 to 39.3]) | 0.80 | 0.84 |
| CPR, n/n_transfers with fresh embryos_ (%)* | 62/128  (48.4 [39.9 to 57.1]) | 25/76  (32.9 [23.3 to 44.2]) | 33/78  (42.3 [31.9 to 53.5]) | 0.086 | 0.48^ƒ^ |
| Live birth rate, n/n_transfers with fresh embryos_ (%)* | 49/128  (38.3 [30.3 to 44.5]) | 24/76  (31.6 [22.2 to 42.8]) | 26/78  (33.3 [23.8 to 44.5]) | 0.60 | 0.50 |
| Miscarriage rate, n/n_clinical pregnancy_ (%)* | 10/62  (16.1 [8.9 to 27.5]) | 1/25  (4.0 [0.6 to 23.5]) | 7/33  (21.2 [10.5 to 38.3]) | 0.32 | NA |
| Cumulative CPR, n/n_total transfers_ (%) | 90/233  (38.6 [31.0 to 47.0]) | 43/129  (33.3 [24.7 to 45.0]) | 46/121  (38.0 [28.5 to 50.8]) | 0.61 | 0.21^#^ |
| Cumulative CPR, n/n_cycles_ (%) | 90/163  (55.2 [44.9 to 67.9]) | 43/92  (46.7 [34.7 to 63.0]) | 46/94  (48.9 [36.6 to 65.3]) | 0.63 | 0.48^#^ |

Values are quoted as the mean [95% CI] or no./total no. (rate [95%CI] in %). The p value was adjusted for the woman’s age, BMI and current smoking status, the type of COH protocol, and the total dose of rFSH.

ƒ additionally adjusted for the number of fresh embryos transferred

# additionally adjusted for the total number of embryos transferred

^a^ p<0.05 vs. PCOS after Bonferroni correction

^b^ p<0.05 vs. PCOM-only after Bonferroni correction

^c^ p<0.05 vs. with controls after Bonferroni correction

Abbreviations: CPR = clinical pregnancy rate, NA = not applicable, due to low frequencies

ǂ cycles with no embryos obtained on day 2-3 were excluded

* cycles with embryo transfer on day 5-6 and cycles with a “freeze-all” strategy were excluded
